# Risk Factors for Antimicrobial Resistance of *Staphylococcus* Species Isolated from Dogs with Superficial Pyoderma and Their Owners

**DOI:** 10.3390/vetsci9070306

**Published:** 2022-06-21

**Authors:** Cheng-Hung Lai, Yu-Chan Ma, Wei-Yau Shia, Yu-Ling Hsieh, Chao-Min Wang

**Affiliations:** 1 Department of Veterinary Medicine, National Chung-Hsing University, 145 Xingda Road, Taichung 402202, Taiwan; chlai@dragon.nchu.edu.tw (C.-H.L.); vmwyshia@nchu.edu.tw (W.-Y.S.); clps5629@yahoo.com.tw (Y.-L.H.); 2 Master’s Degree Program in Department of Veterinary Medicine, National Chung-Hsing University, 145 Xingda Road, Taichung 402202, Taiwan; owencat0718@gmail.com; 3 Department of Veterinary Medicine, National Chiayi University, 580 Xinmin Road, Chiayi 600023, Taiwan

**Keywords:** superficial pyoderma, *Staphylococcus pseudintermedius*, MRSP, dru type, risk factors, owners

## Abstract

The microbial communities on the skin of dogs include several species of bacteria, which contribute to skin health and disease. *Staphylococcus pseudintermedius*, cultured at high frequency from the skin of dogs, is an opportunistic pathogen causing superficial pyoderma. Effective treatment against *S. pseudintermedius* infections is an important issue in veterinary medicine. However, multiple antibiotic-resistant mechanisms gradually developed by bacteria make treatment more challenging nowadays. Drug-resistant genes may have the chance to be transferred from infected dogs to other staphylococci in humans. The objective of this survey is to investigate the bacterial species that cause canine superficial pyoderma and characterize the antibiotic-resistant profiles and drug-resistant genes of isolated *S. pseudintermedius*. In addition, the possible risk factors causing *S. pseudintermedius* colonizing owners were also evaluated by a questionnaire survey. Sixty-five bacteria were isolated from dogs with superficial pyoderma, which included 47 *S. pseudintermedius* (72.3%), 12 other staphylococci (18.5%), 4 other Gram-positive bacteria (6.2%) and 2 Gram-negative bacteria (3.1%). Strains containing *mec*A and *bla*Z genes showed multiple-drug resistance characteristics. Dogs that received antimicrobial treatment within a recent month were at significantly higher risk of MRSP infections. Only five *S. pseudintermedius* strains (8.33%) were isolated from 60 samples of owners. Risk factor analysis indicated there was no significant association between *S. pseudintermedius* isolated from dogs and owners, but the “Keeping three or more dogs” and “Dogs can lick the owner’s face” have high odds ratios of 3.503 and 5.712, respectively. MRSP isolates belonged to three different *dru* types, including *dt11y* (29.41%), *dt11a* (47.06%) and *dt10cp* (23.53%). In conclusion, the major pathogen of canine superficial pyoderma is found to be *S. pseudintermedius* in Taiwan, and isolates which are *mec*A- or *bla*Z-positive are generally more resistant to commonly used antibiotics. Although *S. pseudintermedius* isolated from the owners might be transferred from their dogs, definite risk factors should be examined in the future study.

## 1. Introduction

In clinical cases in dogs, diseases related to the skin are very important. In general, the normal skin of dogs has a variety of defense mechanisms against foreign pathogens at the outermost periphery of the entire body. In addition to physical and chemical barriers, the regular microbial flora on the skin surface is also an important protective layer for the maintenance of healthy skin. The microbial flora with a wide diversity can maintain the balance of the entire microenvironment. They can actively secrete certain substances with antibiotic properties to inhibit the proliferation of foreign microorganisms or perform a scavenging effect [1,2]. However, even with multiple layers of protection, if the dog has a primary or secondary bacterial infection caused by local wounds, skin parasitic infection, or sebum leakage, it will cause a variety of skin lesions. In bacterial infections of the skin, superficial pyoderma is the most common disease in dogs [3].

Superficial pyoderma in dogs refers to bacterial infections involving the dog’s epidermis and epithelium at the hair follicles and can be further subdivided according to the site of bacterial infection. For example, bacterial infections that occur in the hair follicles are called superficial bacterial folliculitis (SBF), while bacterial infections that occur around the mouth or on the lips are called mucocutaneous pyoderma [3]. In dogs, SBF is more common than in other mammalian species and is usually caused by *S. pseudintermedius* [4]. Routine treatment with systemic antimicrobial agents has increased the multi-resistant bacteria, particularly methicillin-resistant *S. pseudintermedius* (MRSP). In staphylococci, resistance to beta-lactam antibiotics, such as methicillin, occurs due to the acquisition of a mobile gene segment called the Staphylococcal cassette chromosome mec (SCCmec) [5]. In SCCmec, the *mec*A gene enables bacteria to produce a “penicillin-binding protein 2a” (PBP2a) that is different from the normal penicillin-binding protein, making bacteria resistant to methicillin. The increasing frequency of multidrug resistance of MRSP complicates the selection of antimicrobial therapy in veterinary medicine.

In addition to the skin of dogs, *S. pseudintermedius* can also be isolated from other organs or systems. However, it can hardly be isolated from healthy humans. *S. pseudintermedius* isolates from humans have been found to be associated with frequent contact between their own dogs, so this species has been regarded as an important zoonotic pathogen in recent years [6,7,8]. In addition to the direct transmission of *S. pseudintermedius* to dog owners, the drug-resistant gene fragments carried by the strain also have the opportunity to exchange with other staphylococci in humans [7,8]. The risk of horizontal gene transfer from the dog to owner strain may induce more multi-drug-resistant bacteria in the future, eventually making it difficult for humans to treat bacterial infections.

As mentioned above, there were fewer studies focusing on risk factors analysis of Staphylococcus species isolated from dogs with superficial pyoderma and their owners. Therefore, this study collected samples from dogs with superficial pyoderma and their owners at the Veterinary Medical Teaching Hospital, Department of Veterinary Medicine, National Chung-Hsing University from 2017 to 2018. In addition to the isolation of canine pathogens, the drug resistance profiles of *S. pseudintermedius* isolates, the detection of drug resistance genes *mec*A and *bla*Z, and the risk factors for MRSP infection in dogs were also investigated. The detection rate of *S. pseudintermedius* will be confirmed in the owner’s samples, and the questionnaire results will be combined to explore the possible risk factors for their owners. Finally, *dru* gene type of the isolated MRSP will also be classified in order to understand the type of strains in Taiwan currently and compare the differences with foreign countries.

## 2. Materials and Methods

### 2.1. Clinical Cases Collection

From 2017 to 2018, dogs presenting superficial pyoderma were identified by a veterinarian at the Veterinary Medical Teaching Hospital, Department of Veterinary Medicine, National Chung-Hsing University. Sixty cases, including skin swabs from dogs and nasal swabs of owners, were submitted to the laboratory for microbiological analysis.

### 2.2. Questionnaire Survey

A survey was designed as a 12-question questionnaire for the owners to fill out. The content of the questions is related to the basic information of the owners and mainly focuses on the interaction between the dogs and the owners. The questionnaire data were further applied for statistical analysis together with the bacterial survey results in the follow-up.

### 2.3. Bacterial Isolation and Identification

The skin and nasal swabs were submitted to the microbiology laboratory within 12 h for bacterial isolation and identification. Swabs were cultured on Columbia agar with 5% sheep blood (BD, Heidelberg, Germany) at 37 °C for 24 to 48 h. Suspected colonies were picked and stained with Gram stain. Gram-positive coccus was subcultured for catalase and coagulase biochemical testing. Genomic DNA was extracted from bacterial samples using a commercial kit (GenoMaker, Blossom Biotech, Inc., Taiwan). 16S rDNA sequencing was used for bacterial identification. 16S rDNA was amplified by 27F and 1492R primer pair and sequences were blasted with the NCBI BLAST database (https://blast.ncbi.nlm.nih.gov/Blast.cgi; accessed on 11 May 2018) according to the references [9,10]. *Staphylococcus intermedius* group (SIG) was further distinguished as *S. intermedius*, *S. pseudintermedius*, and *S. delphini* groups A and B by targeting the nuc gene locus using multiplex PCR [11].

### 2.4. Antimicrobial Susceptibility Testing

According to the CLSI (Clinical and Laboratory Standards Institute) standard method [12], the disk diffusion method was selected for antimicrobial susceptibility testing. Ten antimicrobial agents, including AMC30 (amoxicillin 20 μg + clavulanic acid 10 μg), AMP10 (ampicillin 10 μg), KZ30 (cephazolin 30 μg), CL30 (cephalexin 30 μg), DA2 (clindamycin 2 μg), DO30 (doxycycline 30 μg), ENR5 (enrofloxacin 5 μg), CN10 (gentamycin 10 μg), OX1 (oxacillin 1 μg), P10 (penicillin 10 units), were used in this study. MRSP or MSSP was identified according to the CLSI oxacillin standard [12].

### 2.5. PCR Detection of blaZ and mecA Gene from S. pseudintermeidus

The condition of blaz and mecA gene detection was described as follows: The PCR reaction mixtures contained 100 ng chromosomal DNA, oligonucleotide primers (10 pmols), and 2X Taq DNA Polymerase Mastermix-Red^®^ (Ampliqon, Denmark) at a final volume of 20 μL. The PCR condition for the *blaZ* gene was designed as an initial denaturation step (94 °C, 2 min), 30 cycles of denaturation (94 °C, 1 min), annealing (52 °C, 1 min), and extension (72 °C, 1 min) step, and a final extension step (72 °C, 5 min). The primer pairs used for PCR experiments include the forward primer blaZ F (5′-AAGAGATTTGCCTATGCTTC-3′) the reverse primer blaZ R (5′-GCTTGACCACTTTTATCAGC-3′); the product size was 512 base pairs [13]. The PCR condition for *mec*A gene was designed as an initial denaturation step (94 °C, 4 min), 35 cycles of denaturation (94 °C, 60 s), annealing (55 °C, 60 s), and extension (72 °C, 60 s) step, and a final extension step (72 °C, 10 min). The primer pairs used for mecA detection included the forward primer mecA F (5′-GTAGAAATGACTGAACGTCCGATAA-3′), the reverse primer mecA R (5′-CCAATTCCACATTGTTTCGGTCTAA-3′); the final product size was 310 base pairs [14].

### 2.6. Dru Gene Typing

The condition of *dru* gene typing was described as follows: The PCR reaction mixtures contained 100 ng chromosomal DNA, oligonucleotide primers (10 pmols), 2X Taq DNA Polymerase Mastermix-Red^®^ (Ampliqon, Denmark) at a final volume of 20 μL. The PCR condition for the *dru* gene was designed as an initial denaturation step (95 °C, 5 min), 30 cycles of denaturation (94 °C, 45 s), annealing (52 °C, 45 s), and extension (72 °C, 60 s) step, and a final extension step (72 °C, 5 min). The primer pairs used for PCR experiments included the forward primer dru GF (5′-GTTAGCATATTACCTCTCCTTGC-3′), the reverse primer dru GR (5′-GCCGATTGTGCTTGATGAG-3′), and the product size was about 900 base pairs. The PCR product was further sequenced and the sequence was compared with the data bank (http://dru-typing.org; accessed on 11 May 2018) for *dru* gene typing.

### 2.7. Statistical Analysis

Statistical analysis and charting of data were performed using SAS 9.4 (SAS Institute Inc., Cary, NC, USA) and Excel 2010 (Microsoft, Washington, WA, USA). The Chi-squared test was used to compare whether the presence or absence of drug resistance genes (*mec*A or *bla*Z) and the *dru* typing were related to the resistance of the isolated strains to antibiotics. The questionnaire data were analyzed with the bacterial results using the Chi-squared test and odds ratio (OR). If the expected value in the Chi-square test is less than 5, use Fisher’s Exact Test for statistical analysis. The statistical result was expressed as *p*-values and was considered statistically significant when *p* < 0.05.

## 3. Results

### 3.1. Bacterial Identification in Dogs with Superficial Pyoderma and Their Owners

**Figure 1:**
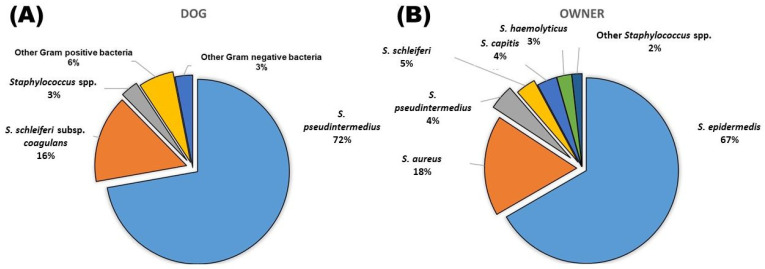
Bacterial isolation and identification from dogs with superficial pyoderma (**A**) and their owner (**B**).

In this study, swabbed samples from the lesion areas of 60 dogs with superficial pyoderma were collected from the Veterinary Teaching Hospital of National Chung-Hsing University, in addition to nasal swabbed samples from the owners of these 60 dogs. A total of 65 strains were isolated from dogs, including 47 strains of *S. pseudintermedius* (72.3%), 10 strains of *S. schleiferi* subsp. *coagulans* (15.4%), 2 strains of other staphylococci (3.1%), 4 strains of other Gram-positive bacteria (6.2%) and 2 strains of Gram-negative bacteria (3.1%) (Figure 1). Two strains were identified as *S. epidermedis* and *S. hominis* in the other staphylococci group. The remaining four Gram-positive bacteria included *Enterococcus gallinarum*, *Enterococcus faecalis*, *Streptococcus halichoeri* and *Streptococcus sanguinis*, respectively. In addition, Gram-negative bacteria were identified as *Sphingonas mucosissima* and *Acinetobacter schindleri*, respectively.

In terms of owners, a total of 114 Staphylococcus strains were isolated, including 76 strains of *S. epidermedis* (66.7%), 20 strains of *S. aureus* (17.5%), 5 strains of *S. pseudintermedius* (4.4%), 4 strains of *S. schleiferi* (3.5%), 4 strains of *S. capitis* (3.5%), 3 strains of *S. haemolyticus* (2.6%), and 1 strain of each *S. gallinarum* (0.9%) and *S. pasteuri* (0.9%) (Figure 1).

### 3.2. Antimicrobial Susceptibility Testing

#### 3.2.1. Antimicrobial Susceptibility Testing of *Staphylocuccus* Isolated from Dogs

Among the 47 strains of *S. pseudintermedius*, 76.6% of the strains were found to be resistant to ampicillin, 72.34% to penicillin G, 55.32% to doxycycline, 48.94% to gentamicin, 40.43% to clindamycin, 31.91% to enrofloxacin, 25.53% to cephalexin and cephazolin and 21.28% to augmentin. Strains of *S. pseudintermedius* isolated in this study were highly resistant to ampicillin but relatively sensitive to augmentin. In the non-*S. pseudintermedius* Staphylococcus group, 83.33% of the strains were resistant to ampicillin and penicillin G, 50% to clindamycin, 33.33% to cephalexin, 16.67% to doxycycline, gentamicin, cephazolin, augmentin and 8.33% to enrofloxacin. The Chi-square test indicated that *S. pseudintermedius* had significant resistance to gentamicin and doxycycline compared to other Staphylococcus strains isolated from the skin of dogs (Table 1, *p* < 0.05).

In multi-drug resistant analysis, 70.21% of the *S. pseudintermedius* strains and 83.33% of the other staphylococcus strains were multi-resistant, respectively. There was no statistically significant difference between these two groups (*p* = 0.581).

#### 3.2.2. Correlation between Antibiotics Resistant Profiles and mecA Gene of *S. pseudintermedius* from Dogs

All *S. pseudintermedius* isolates were submitted for *mec*A gene detection. Sixteen strains were found to be *mec*A positive, and the remaining 31 strains were *mec*A negative. All *mec*A-positive *S. pseudintermedius* strains were found to be resistant to ampicillin and penicillin G, 93.75% to clindamycin, 87.5% to doxycycline and enrofloxacin, 81.25% to gentamicin and oxacillin, and 75% to cephalexin and cephazolin and 62.5% to augmentin. In mecA-negative *S. pseudintermedius* group, 64.52% of the strains were found to be resistant to ampicillin, 38.71% for doxycycline, 32.26% for gentamicin, 18% for penicillin G, 12.9% for clindamycin and oxacillin, and 3.23% for enrofloxacin. It was found that *mec*A-negative *S. pseudintermedius* strains were sensitive to augmentin, cephalexin and cephazolin, respectively. In addition, all of the *mec*A-positive strains were MRSP. The Chi-square test indicated that the presence or absence of *mec*A gene significantly influences the profile of antibiotic resistance in *S. pseudintermedius* (Table 2. *p* < 0.05).

#### 3.2.3. Correlation between Antibiotics Resistant Profiles and *bla*Z Gene of *S. pseudintermedius* from Dogs

All *S. pseudintermedius* isolates were submitted for *bla*Z gene detection; 38 strains were found to be *bla*Z positive, and the remaining nine strains were *bla*Z negative; 94.74% of *bla*Z -positive *S. pseudintermedius* strains were found to be resistant to ampicillin, 89.47% to penicillin G, 68.42% to doxycycline, 60.53% to clindamycin, enrofloxacin, and gentamicin, and 31.58% to cephalexin and cephazolin and 26.32% to augmentin. It was found that *bla*Z-negative *S. pseudintermedius* strains were sensitive to all tested antibiotics. The Chi-square test indicated that the presence or absence of *bla*Z gene significantly influences the gentamicin, clindamycin, ampicillin, doxycycline, penicillin G and enrofloxacin resistance in *S. pseudintermedius* (Table 3. *p* < 0.05).

In addition, most of the *bla*Z-positive *S. pseudintermedius* strains (86.84%) were multi-drug resistant. The chi-square test indicated that the presence or absence of *bla*Z gene significantly influences the multi-drug resistance in *S. pseudintermedius* (Table 3. *p* < 0.0001).

#### 3.2.4. Antibiotics Resistant Profiles and *dru* Gene Typing of *S. pseudintermedius* from Dogs

A total of 17 MRSP strains were isolated in this study, of which 16 strains were from dogs and only one strain was from the owner. The *dru* genes of the 17 MRSP strains were amplified, sequenced and compared with an online database to determine the *dru* gene types. There were three types of *dru* genes found in 17 MRSP strains, including eight strains of dt11a, five strains of dt11y and four strains of dt10cp. In the statistical analysis, the results showed that there was no significant correlation between antibiotic resistance and *dru* gene typing (Table 4).

### 3.3. Risk Factors Analysis

#### 3.3.1. Risk Factors Analysis of MRSP and MSSP from Dogs

In the questionnaire survey, two questions were asked about “whether the owner works in a medical institution” and “whether the dog has received any form of antibiotic treatment within a month”. Of the 45 dogs with *S. pseudintermedius* infection, 16 strains were MRSP and 29 were MSSP. The Chi-square test indicated that the association of owners’ workplace with MRSP or MSSP isolated from dogs in the medical facility had no significant association (*p* = 0.608). However, dogs who received antibiotic treatment within one month had a statistical correlation (*p* = 0.0004) between the isolation of MRSP and MSSP from dogs, with an odds ratio of 11.5 (Table 5). Results indicated that dogs who had been treated with antibiotics within one month had a significantly higher isolation rate of MRSP.

#### 3.3.2. Risk Factors Analysis for *S. pseudintermedius* Isolated from Owners

In this study, only five *S. pseudintermedius* strains were isolated from the nasal cavity of sixty dog owners. The results showed that the dogs raised by the owners who had *S. pseudintermedius* isolated also had *S. pseudintermedius* isolated in the skin lesion, and the detection results of the *mec*A and *bla*Z genes of these five pairs of *S. pseudintermedius* strains were consistent, respectively. However, the results of antibiotic susceptibility tests were slightly different among the five groups of strains (Table 6). Results showed *S. pseudintermedius* strains isolated from dogs generated more antibiotic resistant profiles than the strains isolated from humans, especially for resistance to doxycycline (C9 versus H9) and gentamycin (C41 vs. H41; C45 vs. H45). In addition, strain H35 did not resist any antibiotics tested in this study.

There are 10 questions in the questionnaire about the relationship between the owner and the dog, including “whether there are more than 3 dogs at home”, “whether the dogs are kept indoors”, “whether the dogs can rest on the sofa or seat in the living room”, “whether the dogs can enter the owner’s bedroom, “whether the dogs can rest or be active in the owner’s bedroom for a long time”, “whether the dogs can move on the owner’s bed”, “Contact with dogs more than three times a day”, “whether the dogs can lick owner’s hands”, “whether the dogs can lick owner’s face”, and “whether the dogs can bathe in owner’s bathroom”. The correlation is analyzed by a Chi-square test and the odds ratio at the same time for the above 10 questions and whether *S. pseudintermedius* was isolated from the owner. Results indicated that there was no statistical relationship between the *S. pseudintermedius* isolated from the owner and any item in the questionnaire, but the “Keeping three or more dogs” and “Dogs can lick the owner’s face” have high odds ratios of 3.503 and 5.712, respectively (Table 7).

## 4. Discussion

Superficial pyoderma is a superficial infection by bacteria, such as *S. pseudintermedius*, *S. schleiferi*, *Escherichia coli*, and species of the genera *Pseudomonas* and *Proteus*. *S. pseudintermedius* is a species that has only been identified in the past ten years, and it was often identified as another species of *Staphylococcus* due to insufficient development of classification technology [15,16]. *S. pseudintermedius* is a member of the *S. intermedius* group, which also includes *S. intermedius* and *S. delphini*. The species in this group have similar biochemical properties and high 16s rDNA sequence similarity [17]. According to the evolution of molecular biology, there are various tools that can assist in the identification of *S. pseudintermedius* species and even further classify them according to their genotypes [18,19]. The results of genotyping can be used in epidemiological surveillance and improve the medical care of dogs and their owners.

Previous studies showed that Staphylococcus was the main species isolated from dog skins and most of them can be identified as *S. pseudintermedius* [20,21]. A one-year study in Australia found that 70.8% of the bacteria isolated from companion animals were *S. pseudintermedius* [21]. Another study in South America from 2007 to 2012 found that staphylococci were isolated in 26.5% of samples from various body parts of dogs, and 71.7% were identified as *S. pseudintermedius* [20]. According to the results of this study, *S. pseudintermedius* had the highest rate (72.3%) in dogs with superficial pyoderma, indicating that *S. pseudintermedius* plays an important role in the skin infection of dogs.

Among staphylococci, the most frequently isolated species from human nasal mucosa have been reported to be *S. epidermidis* and *S. aureus*, but the composition ratio varies from person to person [22]. In this study, only 4.4% of the isolates in the owner’s survey were identified as *S. pseudintermedius*. Compared with previous reports, the rate of *S. pseudintermedius* isolated from the nasal cavity of ordinary humans is much higher, probably because the humans in this survey are all dog owners. Many studies have also shown that dog owners have a higher chance of severing SIG or *S. pseudintermedius*, indicating the risk factors of pathogens transfer from pets to owners [23,24].

In this study, *S. pseudintermedius* was significantly more resistant to doxycycline and gentamicin than the rest of the isolated staphylococci among the tested antibiotics. These two antibiotics, doxycycline and gentamicin, are not the first-line treatment for dogs with superficial pyoderma in most veterinary hospitals in Taiwan. Our results may reflect two facts: First, relative to other staphylococci, in facing the treatment of *S. pseudintermedius*, there is a more frequent treatment with non-first-line drugs. Second, *S. pseudintermedius* is a pathogen that is often isolated from canine infections (whether skin or other organs and systems) [25]. Therefore, *S. pseudintermedius* exposure to more diverse antibiotics is predictable. The bacteria have more opportunities to screen out individuals with resistance to multiple antibiotics and cause clinical treatment challenges finally.

According to the results, a total of 47 strains of *S. pseudintermedius* were isolated from the skin lesions of dogs, among which 16 strains carried the *mec*A gene. The proportion of *mec*A-positive *S. pseudintermedius* resistant to the 10 antibiotics tested in this survey was significantly higher than that of *mec*A-negative strains. Interestingly, the 16 *mec*A-positive strains were all multi-drug resistant strains, indicating the presence of *mec*A was significantly correlated with multi-resistant characteristics of *S. pseudintermedius*. *S. pseudintermedius* with the mecA gene can be identified as MRSP because of its resistance to β-lactam antibiotics, such as methicillin. Although different SCCmec species have been reported to have different degrees of multi-drug resistance, most research studies agree that MRSP is related to strains with multi-drug resistance, which is consistent with the findings of this study [26,27,28].

In the results of the *bla*Z gene analysis, most strains of *S. pseudintermedius* were *bla*Z positive and resistant to gentamicin, clindamycin, ampicillin, doxycycline, penicillin G and enrofloxacin. Interestingly, the *bla*Z-negative strains were all susceptible to the antibiotics tested in this study. The possible reason for this phenomenon is *bla*Z-negative *S. pseudintermedius* is more sensitive to commonly used first-line penicillin antibiotics than *bla*Z-positive strains, so it has less chance to encounter other antibiotics and the chance of developing resistance to multiple antibiotics is also reduced.

Questionnaires were also conducted for two items of interest in this survey, namely “Owner works in medical institution” and “Dogs treated with antibiotics within a month”. Originally, speculation had been raised that the owner’s job attributes were prone to encountering severe drug-resistant staphylococci, the interaction between the owner and the dog may lead to the spread of strains, and even further exchange of gene fragments that make bacteria resistant to drugs. However, the results indicated that there was no statistical support for such speculation. A previous study on risk factors for *S. aureus* isolates in dogs and owners showed that colonization of dogs was not associated with close human contact but was strongly associated with health-care occupations [29]. Under the condition that the transmission of strains does exist, the drug resistance gene of Staphylococcus in the owner is likely to be transmitted to the Staphylococcus in dogs. Although this survey has not obtained the predicted results, it may be possible to increase the number of samples and exclude possible interference factors in the future. Moreover, a number of studies indicated that in the dogs who received antibiotic treatment shortly before sampling, the isolation rate of MRSP was significantly increased [30,31,32]. These findings are consistent in this survey and may represent that MRSP has a better survival advantage than MSSP when *S. pseudintermedius* in dogs is under pressure caused by antibiotics, so the possibility of being isolated in dogs is also relatively increased.

Although the probability of *S. pseudintermedius* in dogs being transmitted to humans and causing a direct infection is not high, *S. pseudintermedius* is likely to exchange drug-resistant gene segments with other staphylococci after transmission to humans [23,33,34]. This survey combined questionnaires to clarify whether certain risk factors were associated with the isolation of *S. pseudintermedius* in owners. From the statistical results, it can be seen that “Keeping three or more dogs” and “Dogs can lick the owner’s face” have high odds ratios of 3.503 and 5.712, respectively. A similar study on the statistical analysis of “Keeping three or more dogs” found that it was significantly related to the isolation of *S. pseudintermedius* from the owner [24]. In addition, the high odds ratio of the factor “dogs could lick the owner’s face” suggested that the act of licking the owner’s face may increase the chance of severing *S. pseudintermedius* in the owner. Several studies have shown that MRSP can also be isolated from other healthy dogs and cats living with MRSP-infected dogs and cats [35,36]. Therefore, if the number of dogs raised is greater, the sources of *S. pseudintermedius* transmission may increase for owners, and a higher probability of isolation of the strain from owners can be expected.

In this study, *dru* typing was used to confirm the genetic diversity of the isolated MRSPs in Taiwan. It was found that three types of MRSPs were included in our isolated MRSPs, namely dt11a (47.06%), dt11y (29.41%) and dt10cp (23.53%). All strains of the three types were all multi-drug resistant, and there was no significant correlation between the type and the tested antibiotic resistance. Compared to other countries, it was found that the MRSP isolated in Canada was dominated by four types of dt11a, dt10h, dt9a and dt11af [37]. A comprehensive survey of MRSP isolates in Canada and the United States found that dt11a and dt9a were predominant [38]. Another report also found that dt11a and dt9a were dominant in MRSP collected in Europe and North America [39]. In Asia, the MRSPs isolated from Korea are dt11a and dt11y, while in Thailand they are dt11a and dt11cj, respectively [40,41]. According to the above literature records and our results, dt11a is widely distributed and is the predominant type in the northern hemisphere. Although dt11y also has sporadic been isolated in Europe and the United States, it was identified in South Korea and Thailand in Asia with significantly higher rates of 28.5% and 10.26%. It is considered to be a more prevalent type in Asia. The *dru* type dt10cp, which accounted for about a quarter of our results, was relatively undocumented. This type was first recorded in 2016 and only 7.69% of MRSPs were identified as dt10cp from Thailand [40,41].

This research involves several aspects, from the isolation and identification of skin pathogens from dogs to the transmission of these zoonotic bacteria to owners, and scientific statistical analysis has also been carried out. Although *S. pseudintermedius* isolated from the owners might be transferred from their dogs, definite risk factors should be examined in the future study. Therefore, future research on the *S. pseudintermedius* isolated in Taiwan can use this paper as a stepping stone for more in-depth analysis.

## 5. Conclusions

This research investigates the pathogenic bacteria of superficial pyoderma in dogs, the antibiotics resistant profiles and drug resistance genes of *S. pseudintermedius*, and *dru* gene typing of MRSP in Taiwan. The detection rate of *S. pseudintermedius* isolated from owners and possible risk factors were also statistically analyzed in this survey to provide a possible direction for the prevention of zoonotic transmission of *S. pseudintermedius*. Results showed that strains that contained the *mec*A and *bla*Z gene generated multiple-drug resistance characteristics. Recently antimicrobial treated dogs were at significantly higher risk of MRSP infections. Dru gene typing indicated MRSP isolates belonged to dt11a, dt11y and dt10cp. The dt11y and dt11a were the most commonly detected type of MRSP in Asia. Further studies on definite risk factors should be examined in the future.

## Figures and Tables

**Table 1:** Antibiotics resistant profiles comparing between *S. pseudintermedius* and other *Staphylococcus* spp. from dogs.

Antibiotics	*S. pseudointermedius* (*n* = 47)	Other *Staphylococcus* spp. (*n* = 12)	*p* Value
Gentamicin	48.94% (23)	16.67% (2)	**0.044**
Clindamycin	40.43% (19)	50.00% (6)	0.549
Ampicillin	76.60% (36)	83.30% (10)	1.000
Doxycycline	55.32% (26)	16.67% (2)	**0.017**
Augmentin *	21.28% (10)	16.67% (2)	1.000
Cephalexin	25.53% (12)	33.33% (4)	0.718
Penicillin G	72.34% (34)	83.33% (10)	0.712
Cephazolin	25.53% (12)	16.67% (2)	0.712
Enrofloxacin	31.91% (15)	8.33% (1)	0.151
Multi-drug resistant	70.21% (33)	83.33% (10)	0.581

* Augmentin: amoxicillin + clavulanic acid.

**Table 2:** Antibiotics resistant profiles comparing between *mec*A positive and negative of *S. pseudintermedius* from dogs.

Antibiotics	*mec*A Positive (*n* = 16)	*mec*A Negative (*n* = 31)	*p*-Value
Gentamicin	81.25% (13)	32.26% (10)	**0.002**
Clindamycin	93.75% (15)	12.90% (4)	**<0.0001**
Ampicillin	100.00% (16)	64.52% (20)	**0.009**
Doxycycline	87.50% (14)	38.71% (12)	**0.001**
Augmentin *	62.50% (10)	0.00% (0)	**<0.0001**
Cephalexin	75.00% (12)	0.00% (0)	**<0.0001**
Penicillin G	100.00% (16)	58.06% (18)	**0.002**
Cephazolin	75.00% (12)	0.00% (0)	**<0.0001**
Enrofloxacin	87.50% (14)	3.23% (1)	**<0.0001**
Oxacillin	81.25% (13)	12.90% (4)	**<0.0001**
Multi-drug resistant	100.00% (16)	54.84% (17)	**0.004**

* Augmentin: amoxicillin + clavulanic acid.

**Table 3:** Antibiotics resistant profiles comparing between *bla*Z positive and negative of *S. pseudointermedius* from dogs.

Antibiotics	*bla*Z Positive (*n* = 38)	*bla*Z Negative (*n* = 9)	*p* Value
Gentamicin	60.53% (23)	0.00% (0)	**0.002**
Clindamycin	50.00% (19)	0.00% (0)	**0.007**
Ampicillin	94.74% (36)	0.00% (0)	**<0.0001**
Doxycycline	68.42% (26)	0.00% (0)	**0.0002**
Augmentin *	26.32% (10)	0.00% (0)	0.172
Cephalexin	31.58% (12)	0.00% (0)	0.087
Penicillin G	89.47% (34)	0.00% (0)	**<0.0001**
Cephazolin	31.58% (12)	0.00% (0)	0.087
Enrofloxacin	39.47% (15)	0.00% (0)	**0.041**
Multi-drug resistant	86.84% (33)	0.00% (0)	**<0.0001**

* Augmentin: amoxicillin + clavulanic acid.

**Table 4:** Antibiotics resistant profiles and *dru* gene typing of *S. pseudointermedius* from dogs.

Antibiotics	dt11a (*n* = 8)	dt11y (*n* = 5)	dt10cp (*n* = 4)	*p* Value
Gentamicin	87.5% (7)	60.0% (3)	100.0% (4)	0.394
Clindamycin	87.5% (7)	100.0% (5)	100.0% (4)	1.000
Ampicillin	100.0% (8)	100.0% (5)	100.0% (4)	-
Doxycycline	87.5% (7)	60.0% (3)	100.0% (4)	0.394
Augmentin *	50.0% (4)	60.0% (3)	100.0% (4)	0.344
Cephalexin	62.5% (5)	80.0% (4)	100.0% (4)	0.630
Penicillin G	100.0% (8)	100.0% (5)	100.0% (4)	-
Cephazolin	62.5% (5)	80.0% (4)	100.0% (4)	0.630
Enrofloxacin	100.0% (8)	60.0% (3)	100.0% (4)	0.118

* Augmentin: amoxicillin + clavulanic acid.

**Table 5:** Risk factors analysis of MRSP and MSSP from dogs.

Risk Factors	MRSP from Dogs (*n* = 16)	MSSP from Dogs (*n* = 29)	OR	95% Confidence Interval	*p* Value
Owner works in medical institution	12.5% (2)	6.9% (2)	1.93	0.245–15.185	0.608
Dogs treated with antibiotics within a month	75.0% (12)	20.7% (6)	11.5	2.711–48.777	**0.0004**

**Table 6:** Comparison of *S. pseudintermedius* isolated from dogs and their owners.

Isolated Strains *	*mec*A	*bla*Z	Antibiotics Resistant Profiles **
C9	+	+	CN, DA, AMP, DO, AMC, CL, OX, P, KZ, ENR
H9	+	+	CN, DA, AMP, AMC, CL, OX, P, KZ, ENR
C35	–	+	CN, AMP, DO, P
H35	–	+	–
C41	–	+	CN, AMP, P
H41	–	+	AMP, P
C45	–	+	CN, AMP, P
H45	–	+	AMP, P
C48	–	+	AMP, P
H48	–	+	AMP, P

* The name of isolated strains: C indicates isolated from canine and H is from human. The same strain number means the dog is kept by the same owner. ** AMC: amoxicillin + clavulanic acid, AMP: ampicillin, KZ: cephazolin, CL: cephalexin, DA: clindamycin, DO: doxycycline, ENR: enrofloxacin, CN: gentamycin, OX: oxacillin, P: penicillin.

**Table 7:** Possible risk factors analysis for *S. pseudintermedius* isolated from owners.

Risk Factors	Positive (*n* = 5)	Negative (*n* = 55)	OR	95% Confidence Interval	*p* Value
Keeping three or more dogs	20.0% (1)	10.9% (6)	3.053	0.246–37.892	0.475
Dogs are kept indoors	100.0% (5)	96.3% (53)	-	-	1.000
Dogs can rest on the sofa seat in the living room	100.0% (5)	72.7% (40)	-	-	0.318
Dogs can enter the owner’s bedroom	80.0% (4)	76.3% (42)	1.089	0.108–10.980	1.000
Dogs can rest in the owner’s bedroom for a long time	80.0% (4)	70.9% (39)	1.641	0.170–15.841	1.000
Dogs can move on the owner’s bed	40.0% (2)	47.2% (26)	0.744	0.115–4.805	1.000
Contact with dogs more than three times a day	100.0% (5)	92.7% (51)	-	-	1.000
Dogs can lick owner’s hands	80.0% (4)	70.9% (39)	1.641	0.170–15.841	1.000
Dogs can lick owner’s face	80.0% (4)	38.1% (21)	5.712	0.732–44.556	0.150
Dogs can take a bathe in owner’s bathroom	60.0% (3)	67.2% (37)	0.548	0.071–4.216	1.000

## Data Availability

The data presented in this study are available on request from the corresponding author. The data are not publicly available due to institutional privacy policy.

## Abbreviations

MLST	multi-locus sequence typing
MRSA	methicillin-resistant *Staphylococcus aureus*
MRSP	methicillin-resistant *S. pseudintermedius*
MSSP	methicillin-sensitive *S. pseudintermedius*
PFGE	pulsed-field gel electrophoresis
SCCmec	Staphylococcal cassette chromosome mec
SIG	*Staphylococcus intermedius* group
